# Facial Barotrauma Mimicking Trauma: A Forensic Case of Mask Squeeze in a Diving Fatality

**DOI:** 10.7759/cureus.88839

**Published:** 2025-07-27

**Authors:** Ikuto Takeuchi, Motoo Yoshimiya, Atsushi Ueda, Yu Kakimoto

**Affiliations:** 1 Department of Forensic Medicine, Tokai University School of Medicine, Isehara, JPN

**Keywords:** diving-related death, facial barotrauma, forensic pathology, mask squeeze, periorbital ecchymosis

## Abstract

Periorbital ecchymosis - commonly known as “raccoon eyes” - is a classical indicator of traumatic head injury, particularly basilar skull fractures. However, similar findings may also arise from non-traumatic causes, including systemic disease and barotrauma. We report a rare forensic case of bilateral periorbital ecchymosis caused by facial barotrauma in the absence of external trauma, observed in a fatal skin diving incident. A healthy man in his 20s lost consciousness during the second dive of a recreational session and was recovered from a depth of approximately 20 m. The presence of periorbital ecchymosis initially raised suspicion of head trauma. However, forensic autopsy revealed no skull or facial fractures or intracranial injury. Video footage and scene investigation confirmed the event was a non-violent solo accident. Based on the integration of contextual and pathological findings, the periorbital ecchymosis was attributed to facial barotrauma resulting from an inability to equalize mask pressure after loss of consciousness. This case highlights the importance of recognizing facial barotrauma as a potential mimicker of traumatic injury in water-related deaths. A thorough forensic investigation, including autopsy, scene analysis, and ruling out underlying disease, is essential for accurate diagnosis. Awareness of this mechanism can help prevent misinterpretation and support more reliable medicolegal conclusions.

## Introduction

Periorbital ecchymosis, colloquially known as “raccoon eyes,” is a well-recognized clinical and forensic sign typically associated with basilar skull fractures [1]. The presence of bilateral periorbital bruising in the absence of direct trauma to the orbital region often raises suspicion for underlying cranial injury and prompts further investigation. However, not all cases of periorbital ecchymosis are due to trauma. Various non-traumatic conditions, such as certain systemic diseases or procedural complications, can also produce similar findings [2-4].

One less commonly recognized but clinically relevant mechanism is barotrauma, which refers to tissue injury caused by pressure differentials between internal and external environments [5]. In diving, such injuries can occur in closed air spaces like the lungs, sinuses, and middle ear [5]. When pressure equalization fails during descent, negative pressure can develop, leading to mucosal damage, hemorrhage, or membrane rupture [5]. Although less common, similar mechanisms may also affect facial soft tissues under a diving mask, resulting in localized barotrauma. Failure to equalize the pressure inside a diving mask during descent can lead to negative pressure, which in turn causes soft tissue injury around the eyes-a condition known as "mask squeeze" [6,7].

While facial barotrauma is well-documented in the diving medicine literature [5], reports describing periorbital ecchymosis resulting from mask squeeze are rare, particularly in forensic autopsy settings. Because this finding may resemble signs of external trauma, failure to recognize barotrauma as a potential cause could lead to misinterpretation. A brief discussion of this possibility may be helpful in forensic casework, especially in water-related deaths.

Here, we report a rare forensic case in which bilateral periorbital ecchymosis was ultimately attributed to facial barotrauma sustained during a fatal skin diving incident. This case underscores the importance of considering barotrauma in the differential diagnosis of periorbital ecchymosis, particularly in water-related deaths, and highlights the value of integrating scene context with autopsy findings to ensure accurate medicolegal conclusions.

## Case presentation

A healthy male in his 20s with no known medical history lost consciousness during the second dive of a recreational skin diving session. He had prior experience with skin diving, although his exact level of proficiency was unclear. Video footage confirmed that he remained submerged for approximately 80 minutes before being recovered from a depth of about 20 m. Cardiopulmonary resuscitation was attempted during transport, but he was unresponsive and confirmed dead at the hospital.

The body was refrigerated for two days prior to forensic autopsy. External examination revealed no injuries to the chest, abdomen, back, or extremities. Livor mortis was present on the dorsal surface. No traumatic internal injuries were identified apart from the facial findings described below.

**Figure 1:**
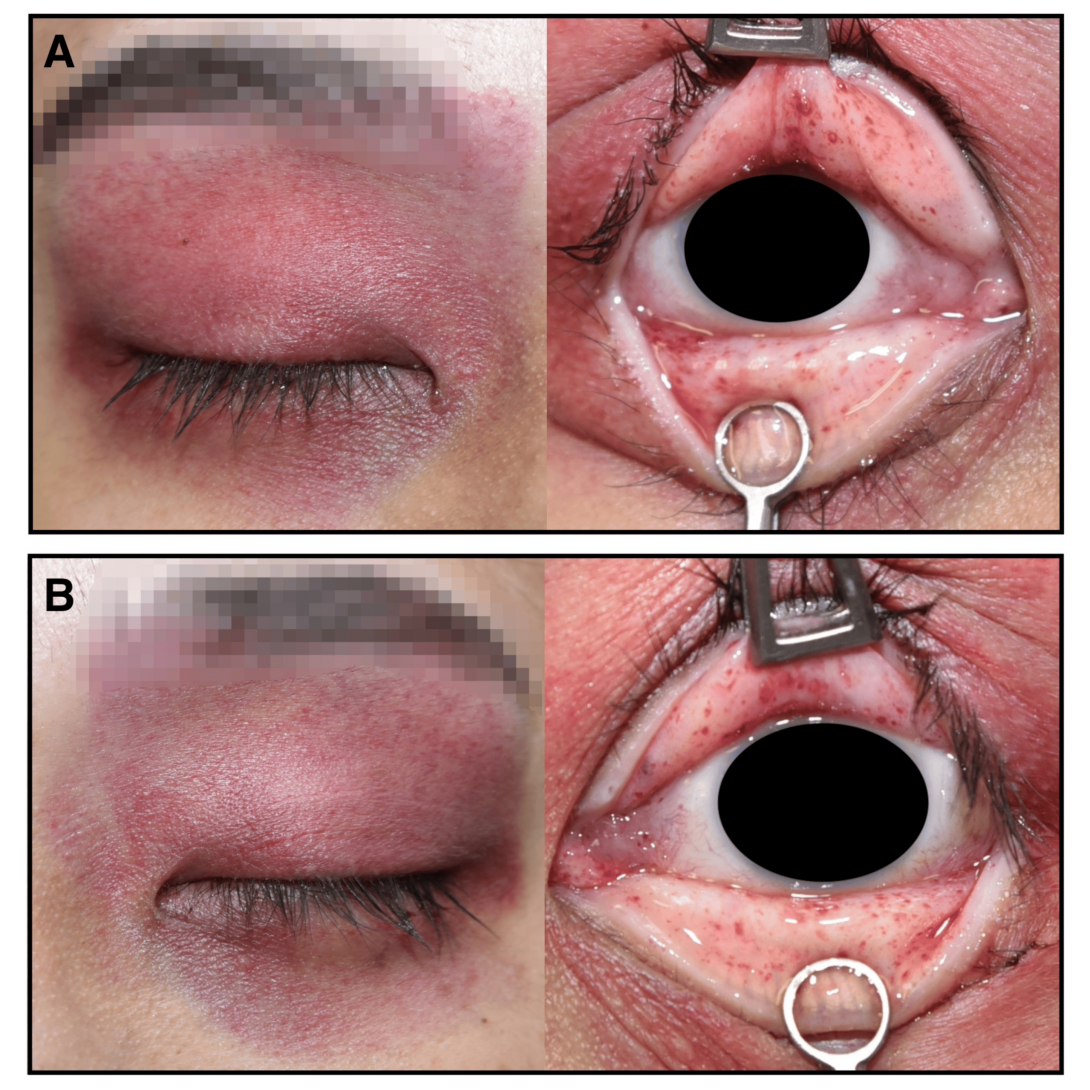
External examination of the periorbital region at autopsy A) Bilateral periorbital ecchymosis is observed, following the contour of the diving mask. Multiple petechial hemorrhages are present on the right upper and lower palpebral conjunctivae. B) Periorbital ecchymosis and petechial hemorrhages are similarly noted on the left upper and lower palpebral conjunctivae. To preserve anonymity, right- and left-sided findings are presented separately in Figure 1A and Figure 1B rather than side by side, and the corneal regions have been obscured digitally.

External examination revealed bilateral periorbital ecchymosis. According to information provided by the police, the discoloration was limited to the area covered by the diving mask. Numerous petechiae were also observed on the upper and lower palpebral conjunctivae (Figures 1A-1B). 

**Figure 2:**
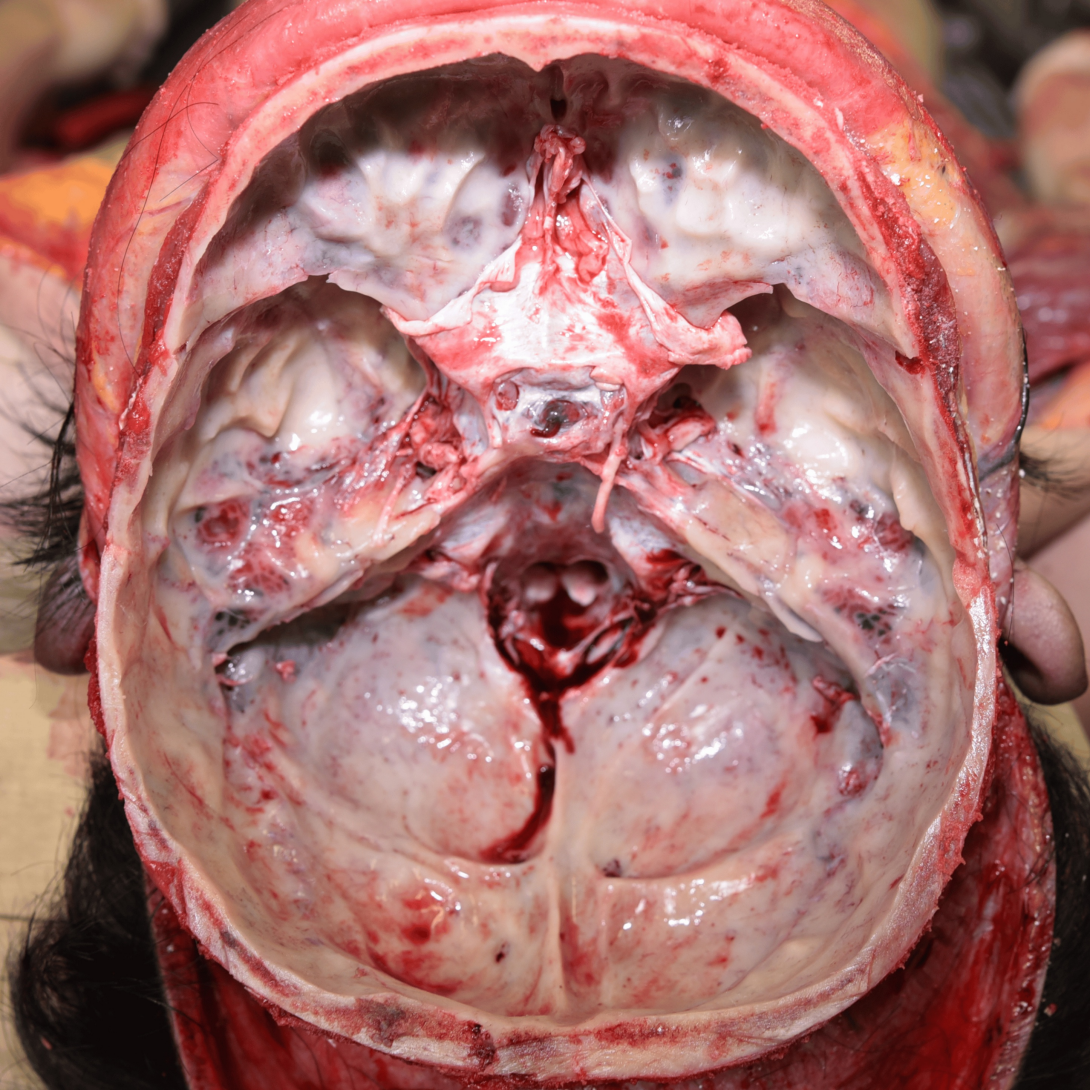
Postmortem examination of the head No fractures of the skull or facial bones were observed on autopsy. There was no evidence of intracranial hemorrhage or other traumatic lesions.

No other facial injuries, such as abrasions or lacerations, were observed. The periorbital ecchymosis was initially suggestive of traumatic head injury, including basilar skull fracture. However, no fractures of the skull or facial bones, nor any intracranial hemorrhage or other traumatic lesions, were identified on autopsy (Figure 2).

**Figure 3:**
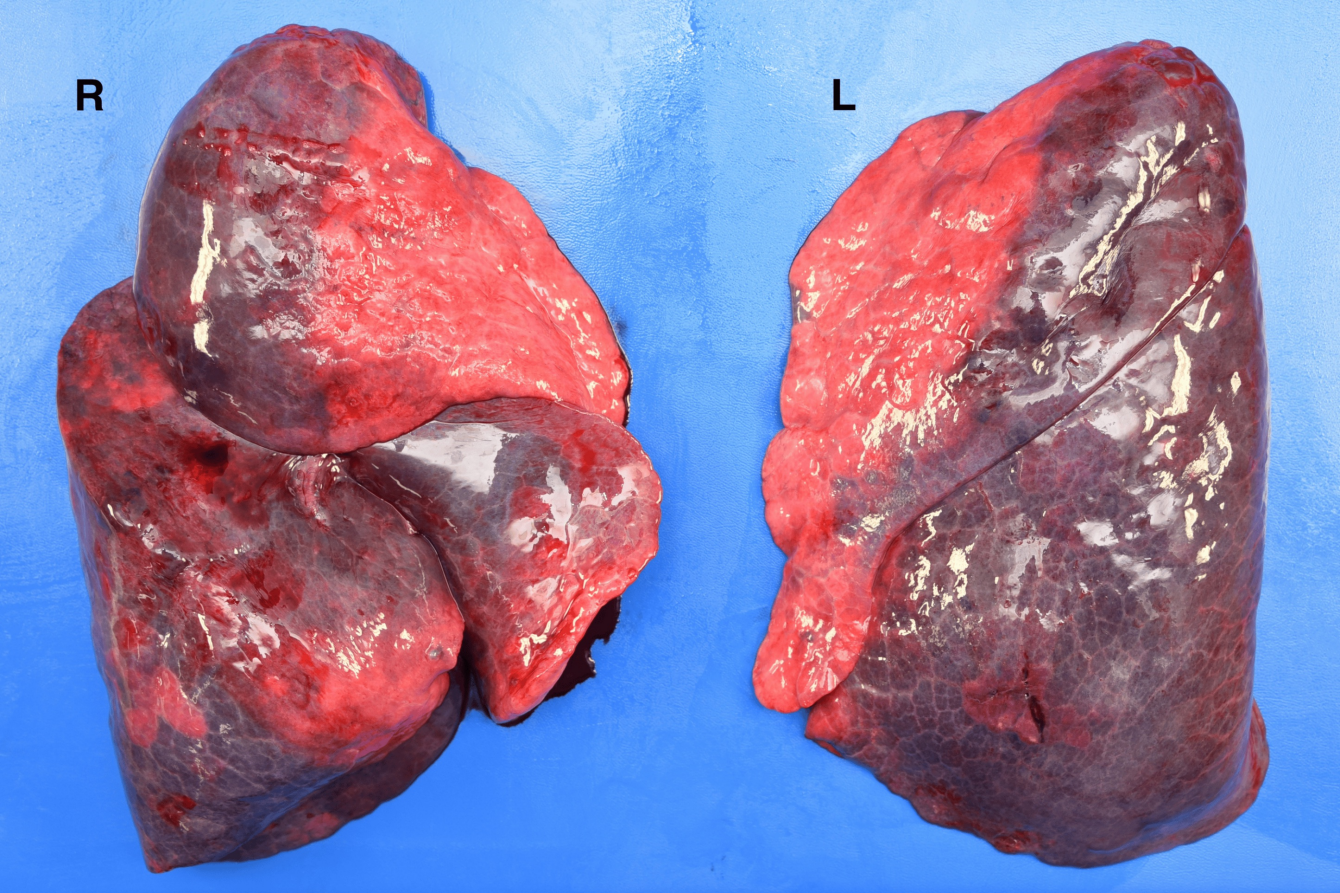
Gross appearance of the lungs at autopsy Both lungs were markedly overdistended, voluminous, and edematous. The lung surfaces appeared smooth and glistening, with increased weight and frothy fluid visible on sectioning. L, left; R, right

In addition to the ocular findings, pulmonary examination revealed markedly overdistended lungs that were voluminous and edematous (Figure 3).

**Figure 4:**
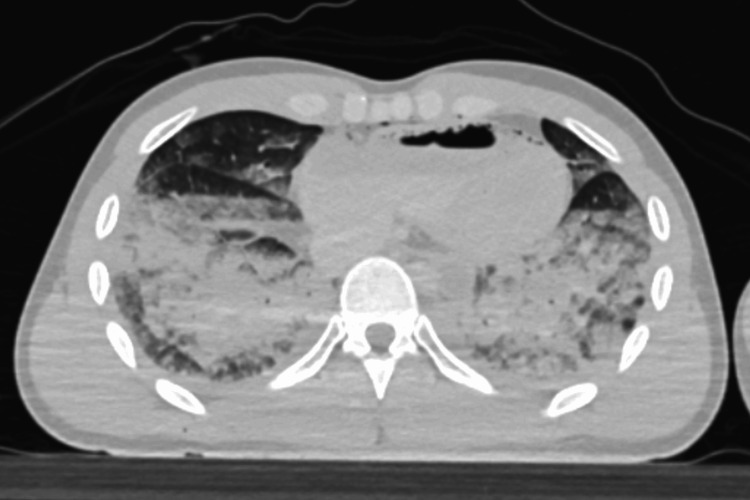
Postmortem CT imaging of the lungs Axial non-contrast postmortem CT image shows diffuse ground-glass opacities and fluid accumulation in both lungs, consistent with pulmonary edema.

Furthermore, non-contrast postmortem CT imaging conducted prior to autopsy revealed diffuse ground-glass opacities and fluid accumulation within the lungs, supporting the presence of pulmonary edema (Figure 4).

Notably, emergency response records indicated the presence of frothy fluid around the mouth and nostrils upon recovery, providing further circumstantial evidence of drowning.

Subsequent police investigation revealed video footage of the incident, confirming the event was a solo diving accident with no third-party involvement. Based on the contextual information and postmortem findings, the periorbital ecchymosis was attributed to facial barotrauma caused by failure to equalize mask pressure after loss of consciousness. Toxicological testing, including alcohol and drug screening, returned negative. The cause of death was determined to be asphyxia due to drowning.

## Discussion

**Figure 5:**
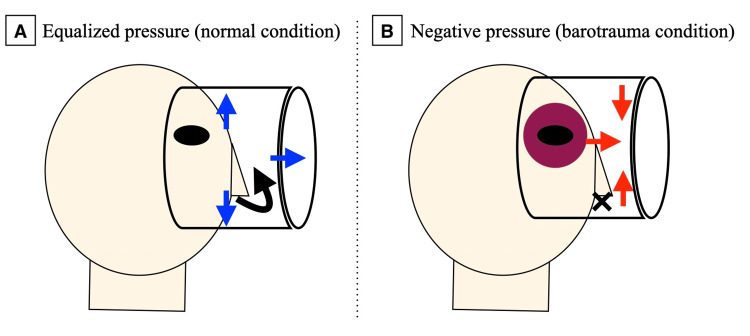
Mechanism of mask squeeze: the role of pressure equalization in facial barotrauma A) Proper exhalation through the nose (black arrow) allows air to enter the mask, equalizing internal pressure (blue arrows) with ambient water pressure. This prevents soft tissue from being drawn inward and protects against facial barotrauma. B) Failure to exhale through the nose during descent results in negative pressure (red arrows) inside the mask. This pressure differential causes the mask to collapse inward, drawing periorbital soft tissue into the mask and leading to periorbital ecchymosis and conjunctival petechiae (purple). This illustration was originally created by the authors based on general anatomical and physiological concepts.

Facial barotrauma is a known complication of diving and typically affects the lungs, middle ear, and paranasal sinuses [5]. When occurring around the mask area, it is often referred to as “mask squeeze” [6], which results from failure to equalize internal mask pressure with ambient water pressure during descent [7]. This condition is normally preventable by exhaling gently through the nose to equalize the pressure [8]. If pressure equalization fails, negative pressure develops within the mask, drawing soft tissue inward and causing rupture of capillaries in the periorbital area and petechial hemorrhages of the conjunctiva (Figure 5).

Although mask squeeze is a recognized complication, reported cases remain limited [6-9], and to our knowledge, no previous forensic autopsy reports have described this finding. This may be due to the fact that diving-related deaths with preserved ocular findings are infrequently autopsied, or that such findings are often overlooked or misattributed to trauma. As such, the present case contributes valuable evidence to the underrecognized forensic manifestations of diving-related barotrauma.

In this case, facial barotrauma likely occurred because the diver lost consciousness and was unable to equalize the mask pressure, allowing further descent or submersion to exacerbate the pressure differential. The characteristic periorbital ecchymosis and conjunctival petechiae were initially suggestive of traumatic head injury. However, comprehensive autopsy findings, including the absence of skull or facial fractures and intracranial hemorrhage, ruled out trauma. Furthermore, video footage from the scene confirmed that the incident was an accidental solo dive with no third-party involvement. Facial congestion is occasionally observed in drowning. However, findings like those in this case are not typically reported in drowning alone. Based on the distribution and appearance, we considered them likely due to negative pressure barotrauma associated with the diving mask.

Bilateral periorbital ecchymosis is typically considered a marker of trauma, especially basilar skull fracture [1]. It is also known to occur in frontal bone fractures [10], orbital floor fractures, and midfacial trauma [11,12], and should initially prompt evaluation for craniofacial injury. Additionally, similar findings have been reported in patients with systemic amyloidosis [2], in whom minor procedures such as endoscopy or renal biopsy can trigger periorbital hemorrhage [3,4]. Therefore, differential diagnosis must also consider systemic disease or non-traumatic invasive triggers.

Distinguishing between non-traumatic (barotrauma), traumatic, and disease-related causes of periorbital ecchymosis requires not only external examination but also a full autopsy and integration of clinical history, medical background, and scene investigation. In the present case, this integrative approach-comprising scene context, visual documentation, and postmortem examination-allowed for a confident determination of non-traumatic facial barotrauma. The deceased was a young, otherwise healthy individual, and routine pathological examination did not reveal any underlying medical conditions.

Additionally, a recent study by Nagar et al. proposed the use of a “drowning index” (DI) - defined as the ratio of the combined weights of both lungs and pleural effusions to the spleen weight - as a quantitative parameter to aid in the diagnosis of freshwater drowning. A DI greater than 9.7 was reported to be highly suggestive of freshwater drowning with a sensitivity of 86.7% and specificity of 70.2% within a postmortem interval of two weeks [13].

In the present case, the left lung weighed 900 g and the right lung 955 g; pleural effusions were approximately 60 g on each side, and the spleen weighed 145 g. This yields a DI of 13.2, well above the proposed diagnostic threshold, thereby providing additional supportive evidence for drowning as the cause of death.

Importantly, this case highlights the potential for facial barotrauma to mimic trauma or assault, particularly in the absence of detailed contextual information. Forensic practitioners should consider facial barotrauma as a differential diagnosis when encountering periorbital hemorrhage in water-related deaths, especially when external trauma is not evident. Awareness of this mechanism can aid in preventing misdiagnosis and ensuring accurate forensic interpretation.

Finally, the approach taken in this case may serve as a reproducible framework for future forensic investigations involving similar findings. By systematically integrating contextual evidence with autopsy results and considering non-traumatic etiologies, forensic pathologists can more accurately assess ambiguous findings and contribute to reliable medicolegal conclusions.

## Conclusions

Facial barotrauma should be considered an important differential diagnosis when periorbital ecchymosis is observed in water-related deaths, particularly in the absence of external trauma or skull fracture. This case illustrates that non-traumatic causes such as mask squeeze can produce findings that closely mimic trauma, highlighting the risk of misinterpretation in forensic settings.

A thorough forensic investigation, including autopsy, scene analysis, and ruling out underlying disease, is essential for accurate diagnosis. In this case, the integration of video evidence, external findings, and pathological assessment enabled a reproducible and reliable determination of facial barotrauma as the underlying mechanism. Raising awareness of this phenomenon can prevent misdiagnosis, improve forensic accuracy, and serve as a valuable educational point for both forensic and clinical practitioners.
